# Supplementary material for: Unveiling the Bioactive Potential of the Invasive Jellyfish Phyllorhiza punctata Through Integrative Transcriptomic and Proteomic Analyses
Source: Biomolecules. 2025 Aug 4;15(8):1121. doi: 10.3390/biom15081121 (PMC12383608; doi:10.3390/biom15081121)
Supplement: Supplementary file 1 [file biomolecules-15-01121-s001.zip › Suplementary Material/FileS3/PEPFOLD4/XP_065058481_5156/clusters.html]

 PEP-FOLD clusters


Download archive of all models  here   

|  |  |  |
| --- | --- | --- |
| # | file | sOPEP |
| # cluster 1 - Size: 1 |  |  |
|  | XP\_065058481\_5156-model1 | -22.8937 |
| # cluster 2 - Size: 43 |  |  |
|  | XP\_065058481\_5156-model2 | -22.4165 |
|  | XP\_065058481\_5156-model2.1 | -22.3684 |
|  | XP\_065058481\_5156-model2.2 | -22.3433 |
|  | XP\_065058481\_5156-model2.3 | -22.2654 |
|  | XP\_065058481\_5156-model2.4 | -22.2608 |
|  | XP\_065058481\_5156-model2.5 | -22.2598 |
|  | XP\_065058481\_5156-model2.6 | -22.252 |
|  | XP\_065058481\_5156-model2.7 | -22.2069 |
|  | XP\_065058481\_5156-model2.8 | -22.2059 |
|  | XP\_065058481\_5156-model2.9 | -22.1955 |
|  | XP\_065058481\_5156-model2.10 | -22.1619 |
|  | XP\_065058481\_5156-model2.11 | -22.1619 |
|  | XP\_065058481\_5156-model2.12 | -22.1619 |
|  | XP\_065058481\_5156-model2.13 | -22.1587 |
|  | XP\_065058481\_5156-model2.14 | -22.1292 |
|  | XP\_065058481\_5156-model2.15 | -22.1292 |
|  | XP\_065058481\_5156-model2.16 | -22.1288 |
|  | XP\_065058481\_5156-model2.17 | -22.1138 |
|  | XP\_065058481\_5156-model2.18 | -22.0688 |
|  | XP\_065058481\_5156-model2.19 | -22.0548 |
|  | XP\_065058481\_5156-model2.20 | -22.0353 |
|  | XP\_065058481\_5156-model2.21 | -22.0281 |
|  | XP\_065058481\_5156-model2.22 | -22.0281 |
|  | XP\_065058481\_5156-model2.23 | -22.0244 |
|  | XP\_065058481\_5156-model2.24 | -22.0244 |
|  | XP\_065058481\_5156-model2.25 | -21.9964 |
|  | XP\_065058481\_5156-model2.26 | -21.9776 |
|  | XP\_065058481\_5156-model2.27 | -21.9709 |
|  | XP\_065058481\_5156-model2.28 | -21.9339 |
|  | XP\_065058481\_5156-model2.29 | -21.9183 |
|  | XP\_065058481\_5156-model2.30 | -21.9183 |
|  | XP\_065058481\_5156-model2.31 | -21.9105 |
|  | XP\_065058481\_5156-model2.32 | -21.8004 |
|  | XP\_065058481\_5156-model2.33 | -21.7596 |
|  | XP\_065058481\_5156-model2.34 | -21.7259 |
|  | XP\_065058481\_5156-model2.35 | -21.6937 |
|  | XP\_065058481\_5156-model2.36 | -21.6408 |
|  | XP\_065058481\_5156-model2.37 | -21.4712 |
|  | XP\_065058481\_5156-model2.38 | -21.3994 |
|  | XP\_065058481\_5156-model2.39 | -21.3489 |
|  | XP\_065058481\_5156-model2.40 | -20.6443 |
|  | XP\_065058481\_5156-model2.41 | -20.4157 |
|  | XP\_065058481\_5156-model2.42 | -19.1398 |
| # cluster 3 - Size: 6 |  |  |
|  | XP\_065058481\_5156-model3 | -19.3877 |
|  | XP\_065058481\_5156-model3.1 | -19.151 |
|  | XP\_065058481\_5156-model3.2 | -19.0296 |
|  | XP\_065058481\_5156-model3.3 | -18.9856 |
|  | XP\_065058481\_5156-model3.4 | -18.8004 |
|  | XP\_065058481\_5156-model3.5 | -18.7102 |
| # cluster 4 - Size: 2 |  |  |
|  | XP\_065058481\_5156-model4 | -18.7911 |
|  | XP\_065058481\_5156-model4.1 | -18.4169 |
| # cluster 5 - Size: 45 |  |  |
|  | XP\_065058481\_5156-model5 | -18.4243 |
|  | XP\_065058481\_5156-model5.1 | -18.3938 |
|  | XP\_065058481\_5156-model5.2 | -18.3938 |
|  | XP\_065058481\_5156-model5.3 | -18.3653 |
|  | XP\_065058481\_5156-model5.4 | -18.3309 |
|  | XP\_065058481\_5156-model5.5 | -18.3019 |
|  | XP\_065058481\_5156-model5.6 | -18.2992 |
|  | XP\_065058481\_5156-model5.7 | -18.2688 |
|  | XP\_065058481\_5156-model5.8 | -18.2474 |
|  | XP\_065058481\_5156-model5.9 | -18.2147 |
|  | XP\_065058481\_5156-model5.10 | -18.2127 |
|  | XP\_065058481\_5156-model5.11 | -18.2095 |
|  | XP\_065058481\_5156-model5.12 | -18.184 |
|  | XP\_065058481\_5156-model5.13 | -18.1774 |
|  | XP\_065058481\_5156-model5.14 | -18.1501 |
|  | XP\_065058481\_5156-model5.15 | -18.1467 |
|  | XP\_065058481\_5156-model5.16 | -18.1428 |
|  | XP\_065058481\_5156-model5.17 | -18.1426 |
|  | XP\_065058481\_5156-model5.18 | -18.1298 |
|  | XP\_065058481\_5156-model5.19 | -18.1272 |
|  | XP\_065058481\_5156-model5.20 | -18.1272 |
|  | XP\_065058481\_5156-model5.21 | -18.1001 |
|  | XP\_065058481\_5156-model5.22 | -18.0661 |
|  | XP\_065058481\_5156-model5.23 | -18.058 |
|  | XP\_065058481\_5156-model5.24 | -18.0539 |
|  | XP\_065058481\_5156-model5.25 | -18.0525 |
|  | XP\_065058481\_5156-model5.26 | -18.038 |
|  | XP\_065058481\_5156-model5.27 | -18.0359 |
|  | XP\_065058481\_5156-model5.28 | -18.0324 |
|  | XP\_065058481\_5156-model5.29 | -18.0307 |
|  | XP\_065058481\_5156-model5.30 | -18.0124 |
|  | XP\_065058481\_5156-model5.31 | -17.9917 |
|  | XP\_065058481\_5156-model5.32 | -17.9763 |
|  | XP\_065058481\_5156-model5.33 | -17.9499 |
|  | XP\_065058481\_5156-model5.34 | -17.9435 |
|  | XP\_065058481\_5156-model5.35 | -17.9298 |
|  | XP\_065058481\_5156-model5.36 | -17.897 |
|  | XP\_065058481\_5156-model5.37 | -17.8357 |
|  | XP\_065058481\_5156-model5.38 | -17.8148 |
|  | XP\_065058481\_5156-model5.39 | -17.7446 |
|  | XP\_065058481\_5156-model5.40 | -17.6311 |
|  | XP\_065058481\_5156-model5.41 | -17.6264 |
|  | XP\_065058481\_5156-model5.42 | -17.3008 |
|  | XP\_065058481\_5156-model5.43 | -17.2984 |
|  | XP\_065058481\_5156-model5.44 | -16.2208 |
| # cluster 6 - Size: 3 |  |  |
|  | XP\_065058481\_5156-model6 | -18.0351 |
|  | XP\_065058481\_5156-model6.1 | -17.9895 |
|  | XP\_065058481\_5156-model6.2 | -17.8738 |
|  |  |  |
